# Migrasomes in cancers: from the biogenesis mechanism to therapeutic implications

**DOI:** 10.20517/evcna.2026.70

**Published:** 2026-09-18

**Authors:** Luomeng Qian, Shoujun Wang, Paulina Kucharzewska, Jiachang Li, Sihe Zhang

**Affiliations:** ^1^Department of Cell Biology, School of Medicine, Nankai University, Tianjin 300071, China.; ^2^Intensive Care Unit, General Hospital of Central Theater Command, Wuhan 430070, Hubei, China.; ^3^Department of Thyroid and Breast Surgery, The Institute of Translational Medicine, Tianjin Cancer Institute of Integrative Traditional Chinese and Western Medicine, Tianjin Union Medical Center, The First Affiliated Hospital of Nankai University, Nankai University, Tianjin 300121, China.; ^4^Center of Cellular Immunotherapies, Warsaw University of Life Sciences, Warsaw 02-787, Poland.; ^#^These authors contributed equally to this work.

**Keywords:** Migrasome, cancer, drug resistance, diagnosis, therapy

## Abstract

Migrasomes are a newly identified class of extracellular vesicles (EVs) with a distinctive pomegranate-like morphology that are generated specifically from migrating cells. They are increasingly recognized as critically important mediators of intercellular communication involved in diverse physiological and pathological processes. However, their roles in cancers remain incompletely defined, and a consensus on migrasome-mediated malignant phenotypes is still lacking. In this review, we first summarize the current knowledge of migrasome biogenesis, distribution, and isolation methodologies. We then systematically discuss the multifaceted roles of migrasomes within the tumor microenvironment (TME), with particular emphasis on their contribution to cancer metastasis, progression and drug resistance. Furthermore, we explore the potential clinical applications of migrasomes in cancer diagnosis and therapy, and outline the key challenges and controversies that hinder their translation into clinical practice. Overall, this review provides an updated perspective on the roles of migrasomes in cancer biology and highlights the emerging opportunities for migrasome-informed targeted therapeutics.

## INTRODUCTION

Migrasomes were first identified in 2015 as vesicles specifically generated by migrating cells; these vesicles are characterized by their distinctive pomegranate-like morphology and formation at the tips or branches of retraction fibers (RFs)^[1]^. These RFs are elongated structures that trail behind migrating cells. The migrasomes gradually enlarge to a size of approximately 0.5-3 μm. Within migrasomes, numerous extracellular vesicle (EV)-like migrasome-derived nanoparticles (MDNPs), typically 50-100 nm in diameter, can be observed. Exosomes generally originate from the endosomal system through inward budding of multivesicular bodies (MVBs)^[2]^. In contrast, migrasomes form via a unique mechanism that involves the abrupt disruption of RFs during cell migration^[1,3]^. This process can be divided into three main stages: (1) nucleation mediated by sphingomyelin synthase 2 (SMS2); (2) maturation driven by phosphatidylinositol 4,5-bisphosphate [PI(4,5)P_2_] signaling and integrin recruitment; and (3) expansion stabilized by tetraspanin (TSPAN)-enriched microdomains^[3,4]^. These unique biogenesis mechanisms position migrasomes as specialized vesicles for targeted delivery of cellular contents during cell migration.

Accumulating evidence indicates that migrasomes play crucial roles in various biological processes, including embryonic development, immune response, wound healing, and tissue regeneration^[5,6]^. They have also been implicated in the pathogenesis of multiple diseases, including viral infections, cardiovascular disorders, neurological diseases, and cancers^[7,8]^. Given that migrasome formation is intrinsically linked to cell migration, understanding the role of migrasomes in cancer biology is essential. Recent studies have highlighted the multifaceted role of migrasomes in cancer progression. Migrasomes have been shown to mediate intercellular communication within the tumor microenvironment (TME), facilitating the interaction between tumor cells and stromal components, including immune cells and mesenchymal stem cells^[9]^. Furthermore, migrasomes contribute to tumor metastasis by transferring signaling molecules to distant sites^[10]^. Emerging evidence also suggests that migrasomes contribute to cancer drug resistance, potentially by transferring drug-resistant molecules to recipient cancer cells^[11]^.

Despite these advances, the exact function of migrasomes in cancers remains incompletely understood. Several critical questions remain to be addressed: how exactly do migrasomes influence cancer metastasis and progression within the TME? What molecular mechanisms underlie migrasome-mediated drug resistance? Can migrasomes serve as reliable diagnostic biomarkers or therapeutic targets in cancers? Furthermore, can all cell types produce migrasomes, and can all cells take up or interact with migrasomes? Do migrasomes from different cell types uniformly influence cancers and do all cancer-derived migrasomes have similar functions? A comprehensive systematization of current knowledge is essential to address these questions and to guide future research. To better understand migrasome-induced malignant phenotypes, this review systematically summarizes the research progress on migrasomes, highlights their role in cancer pathology, and discusses their potential clinical applications in cancer diagnosis and therapy.

## BIOGENESIS AND FATE DISTRIBUTION OF MIGRASOMES

As cells migrate, migrasomes are left behind along RFs. Migrasomes differ from other EVs, such as exosomes and oncosomes, in terms of diameter, morphology, biogenesis, biomarkers, isolation methods and functions in cancers [Table 1]^[1-3,9,10,12-33]^. Migrasomes are formed through a multistep process that includes nucleation, maturation, and expansion stages [Figure 1].

**Figure 1:**
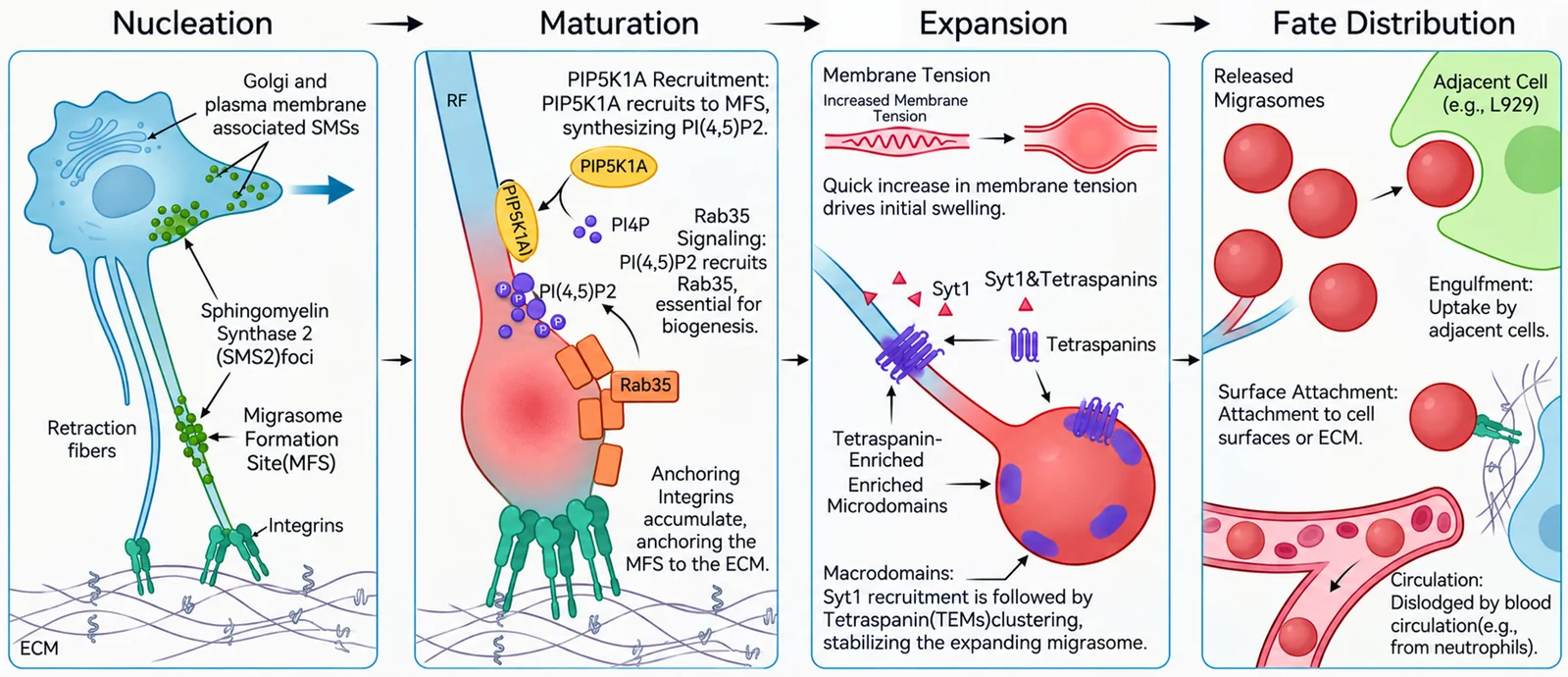
Biogenesis and fate of migrasomes. Migrasome formation is a multistage process comprising nucleation, maturation, and expansion stages. SMS2 initiates nucleation at migrasome formation sites by converting ceramide into SM, thereby promoting local membrane curvature. PIP5K1A and Rab35 subsequently facilitate the recruitment of integrins, establishing adhesion to the ECM. During maturation, tetraspanins and Syt1 regulate membrane expansion and stabilization, leading to the formation of TEMs that coalesce into mature migrasomes. As RFs disintegrate, migrasomes are released into the extracellular space, where they can be internalized by neighboring cells or persist as extracellular structures, thereby mediating intercellular communication. ECM: Extracellular matrix; MFSs: migrasome formation sites; PI(4,5)P2: phosphatidylinositol 4,5-bisphosphate; PI4P: phosphatidylinositol 4-phosphate; PIP5K1A: phosphatidylinositol 4-phosphate 5-kinase 1 alpha; Rab35: Ras-related GTP-binding protein 35; RFs: retraction fibers; SM: sphingomyelin; SMS2: sphingomyelin synthase 2; Syt1: synaptotagmin 1; TEMs: tetraspanin-enriched microdomains.

**Table 1:** Comparison of cancer-derived migrasomes, exosomes, and oncosomes

**Characteristic**	**Migrasomes**	**Exosomes**	**Oncosomes**
Diameter	500-3,000 nm	30-150 nm	500-5,000 nm
Feature	Pomegranate-like morphology containing small nanoparticles	Nano-scale extracellular vesicles secreted by almost all the cells^[15]^	Amorphous, arising from amoeboid blebbing
Biogenesis	Forming at the tips or branches of RFs	Forming through four steps: MVB formation, cargo sorting to MVBs, transport of MVBs, and fusion of MVBs with plasma membrane	Forming from nonapoptotic cell membrane
Biomarkers	CPQ, NDST1, EOGT, PIGK, Integrins (ITGA5/ITGB1, ITGA1, ITGA3), Tetraspanins (TSPAN4, TSPAN7)^[13,16-18]^	CD9, CD63, TSG101, CD81, Alix, HSP70^[2,19,20]^	Cav1 and Arf6^[2,21-23]^
Isolation method	Centrifugation (17,000 × *g*), reversed filtration; Reverse filtration (0.45 µm); WGA magnetic beads; Cytochalasin B + ficoll^[1,3,10,24]^	Ultracentrifugation (100,000-200,000 g), SEC, precipitation-based methods (e.g., ExoQuick), and filtration^[12,25-27]^	Low-speed centrifugation (2,000-10,000 g)^[2,21,23,28]^
Functions in cancers	Reduce apoptosis, promote proliferation, migration, adhesion, and drug resistance in cancer cells^[9,29,30]^	Promote cancer cell invasion, metastasis, and drug resistance^[2,19,31,32]^	Transfer oncogenic material, promote tumor progression^[2,22,23,33]^

ALIX: ALG-2-interacting protein X; Arf6: ADP-ribosylation factor 6; CD9: cluster of differentiation 9; CD63: cluster of differentiation 63; CD81: cluster of differentiation 81; Cav1: caveolin 1; CPQ: carboxypeptidase Q; EOGT: EGF domain-specific O-linked N-acetylglucosamine transferase; HSP70: heat shock protein 70 kDa; ITGA1: integrin alpha 1; ITGA3: integrin alpha 3; ITGA5: integrin alpha 5; ITGB1: integrin beta 1; MVBs: multivesicular bodies; NDST1: N-deacetylase-N-sulfotransferase 1; PIGK: phosphatidylinositol glycan, class K; RFs: retraction fibers; SEC: size-exclusion chromatography; TSG101: tumor susceptibility gene 101; TSPAN4: tetraspanin 4; TSPAN7: tetraspanin 7; WGA: wheat germ agglutinin.

### Biogenesis

#### Nucleation

Migrasome formation sites (MFSs) are frequently observed at the ends or branch points of RFs. Sphingomyelin (SM), a type of sphingolipid, is essential for migrasome formation. SM is derived from ceramide and synthesized by SMS2 at MFSs. At the leading edge of migrating cells, SMS2 is organized into specialized structures known as SMS2 foci, which are associated with the basal membrane. During cell migration, these SMS2 foci concentrate at the leading edge and are ultimately left behind on RFs, where they serve as sites for migrasome formation^[3,16]^. The early aggregation of SMS2 foci resembles a nucleation event, and represents a foundational step in migrasome biogenesis^[3,16]^.

#### Maturation

Once nucleation is initiated, the PI(4,5)P_2_ is synthesized from phosphatidylinositol 4-phosphate (PI4P) by phosphatidylinositol 4-phosphate 5-kinase (PIP5K)^[16,34]^. This newly synthesized lipid recruits and interacts with Ras-related GTP-binding protein 35 (Rab35), which subsequently recruits integrins^[16]^. Integrins are heterodimeric proteins composed of α and β subunits, and are highly enriched in migrasomes. These integrin heterodimers specifically bind to corresponding extracellular matrix (ECM) proteins. The interaction between integrin heterodimers and ECM proteins is crucial for migrasome maturation^[35]^.

#### Expansion

Following maturation, migrasome biogenesis enters the expansion phase. Recent studies using live cell imaging and biomimetic systems have shown that a rapid increase in membrane tension induces localized swelling of RFs, thereby initiating migrasome expansion^[36]^. The recruitment of synaptotagmin 1 (Syt1), a calcium sensor, promotes migrasome biogenesis through calcium binding. At MFSs, Syt1 induces the swelling of these sites into unstable precursors, which are subsequently stabilized through sequential recruitment of TSPANs. Driven by membrane tension, TSPAN molecules progressively cluster at these expanding membrane protrusions^[37]^. TSPANs are transmembrane proteins characterized by four transmembrane domains and can form tetraspanin-enriched microdomains (TEMs) within the cell membrane. TEMs are essential for migrasome formation^[14]^. These microdomains further assemble into larger TSPAN-enriched macrodomains (TEMAs) through the cooperative clustering of TSPANs and cholesterol. The subsequent membrane tension-mediated expansion of TEMAs drives their transformation into mature migrasomes.

### Fate distribution

Migrasomes are widely distributed across multiple species, including humans, mice, rats, and zebrafish^[1,8,38,39]^. They are frequently observed in cells with strong migratory capacity, such as immune cells and metastatic cancer cells. For example, receptor activator of nuclear factor κB ligand (RANKL) promotes the differentiation of murine macrophages into osteoclasts, and triggers migrasome formation^[40]^. In addition, migrasomes have been found in alveoli and blood vessels^[41]^.

When RFs disintegrate, migrasomes are released into the extracellular space. After release, migrasomes may follow multiple routes. They can be taken up by neighboring cells, as observed in mouse fibroblasts^[5]^. Alternatively, migrasomes may remain at their site of origin or be transported to other regions of the organism. For example, neutrophils generate migrasomes within blood vessels, where they remain transiently before most are displaced by blood flow^[42]^.

The fate distribution of migrasomes is closely associated with their functional roles, which depend on cargo loading and release. Migrasomes exhibit four major functional fates: (1) targeted ligand delivery: migrasomes transport specific ligands to specific locations at specific times, thereby influencing biological processes such as embryonic development^[6,39]^; (2) lateral transfer vesicles: migrasomes released by one cell can be internalized by neighboring cells, facilitating intercellular exchange of materials and information^[1,5]^; (3) cellular quality control: migrasomes contribute to cellular homeostasis by removing damaged organelles, toxic substances, or unnecessary cellular components^[42]^; and (4) molecular absorption platforms: migrasomes can capture extracellular molecules and accumulate components released by surrounding cells on their surface^[43]^. These distinct fates contribute to the diverse biological functions of migrasomes.

## ISOLATION AND CHARACTERIZATION OF MIGRASOMES

### Isolation of migrasomes

#### Cell-derived migrasomes

Four major approaches have been developed for the isolation and purification of migrasomes. The first approach, introduced by Yu’s laboratory, is similar to conventional EV isolation and involves sequential low-speed centrifugation, density-gradient centrifugation, and ultracentrifugation^[1]^. In this protocol, OptiPrep is used instead of sucrose as the density medium, which helps preserve the structural integrity of migrasomes. Second, Liang *et al*. developed a filter-based retention and reverse-filtration elution method that exploits the differences in vesicle size. In this method, samples are passed through a 0.45 µm filter, allowing migrasomes, which are larger than the pore size, to be retained on the membrane. By inverting the filter and washing it with buffer solution, migrasomes can then be efficiently recovered^[3]^. Third, Yang *et al*. introduced an innovative, straightforward, and efficient technique for migrasome isolation via wheat germ agglutinin (WGA)-conjugated magnetic beads combined with flow cytometry, enabling highly effective migrasome enrichment^[24]^. Finally, Gu *et al*. used cytochalasin B to promote the detachment of migrasomes from the cell body. Subsequently, Ficoll density gradient centrifugation was used to isolate migrasomes while maintaining membrane integrity^[10]^. Each of these methodologies offers distinct advantages and technical limitations. The ultracentrifugation-based method utilizing OptiPrep density gradients (Yu’s lab) effectively preserves the migrasome ultrastructure; however, this method is time-consuming and may cause mechanical damage during high-speed centrifugation. The filter-based size-exclusion method developed by Liang *et al*. is operationally simple but may suffer from membrane clogging, limited purity, and mechanical stress during filtration^[3]^. WGA-conjugated magnetic bead isolation coupled with flow cytometry (Yang *et al*.) offers high specificity but requires sophisticated instrumentation and incurs relatively high costs^[24]^. In contrast, cytochalasin B coupled with the Ficoll gradient method (Gu *et al*.) preserves membrane integrity, although chemical treatment may perturb physiological conditions and alter downstream signaling properties^[10]^.

Given the coexistence of migrasomes with exosomes and other EVs in conditioned media, a critical challenge in migrasome isolation is achieving high purity. All current migrasome isolation methods risk the coisolation of contaminants. Ultracentrifugation may pellet apoptotic bodies (1-5 µm), large microvesicles (100-1,000 nm), and protein aggregates alongside migrasomes; density gradient purification partially mitigates but does not eliminate this issue. Filter-based methods are susceptible to membrane clogging, which can trap smaller EVs and create artificial aggregates; reverse filtration may also shear large migrasomes into small fragments. WGA-bead isolation, while specific for glycosylated membrane proteins, may capture WGA-binding exosomes and glycoprotein complexes unrelated to migrasomes. Cytochalasin B treatment can induce nonspecific membrane blebbing, potentially generating artifacts that resemble migrasomes in morphology but differ in molecular composition. To maximize purity, a multistep validation strategy is recommended: (1) preclear the conditioned media of cells and debris by sequential low-speed centrifugation (300 × *g*, 10 min; 2,000 × *g*, 20 min); (2) apply the chosen isolation method; (3) verify migrasome identity via positive markers (TSPAN4, ITGA5, EOGT, *etc*.) and negative markers (CD9, CD63, TSG101, *etc*.); (4) assess morphology via transmission electron microscopy (TEM); and (5) if possible, confirm migration-dependent formation by comparing migrasome yields from migrating *vs*. stationary cells. In addition, not all cell types produce migrasomes efficiently. Migrasome production is intrinsically linked to cell migration capacity; therefore, highly migratory cell lines (e.g., NRK, L929, and certain metastatic cancer cell lines) are preferred for migrasome isolation^[1,5]^. Cells with limited migratory activity (e.g., stationary epithelial cells) produce few migrasomes and are unsuitable for isolation. The timing of conditioned media collection is also critical. For most migrasome-producing cell lines, media should be collected after 24-48 h of culture in migrasome-depleted serum or serum-free conditions to minimize contamination with serum-derived EVs^[1,3]^.

Overall, the choice of isolation method depends on a specific experimental objective, balancing structural preservation, yield, efficiency, and purity.

#### Serum-derived migrasomes

The isolation of migrasomes from serum generally follows procedures similar to those used for cell-derived migrasomes, particularly the protocol established by Yu’s laboratory^[13]^. In addition, Hu *et al*. isolated migrasomes from plasma via differential centrifugation to obtain crude migrasome preparations^[8,44]^. Further methods for isolating migrasomes from body fluids are currently being explored.

### Characterization of migrasomes

#### TEM, LSCM and HIS-SIM

Techniques such as transmission electron microscopy (TEM), scanning electron microscopy (SEM), laser scanning confocal microscopy (LSCM), and high-sensitivity structured illumination microscopy (HIS-SIM) are among the most direct and informative methods for the morphological evaluation of migrasomes^[45,46]^. TEM and SEM provide extremely high resolution, enabling visualization of the ultrastructural features of migrasomes. Numerous round vesicle-like structures have been observed along the distal regions of RFs, resembling strings of beads that may either remain connected or exist separately. LSCM enables visualization of fluorescently labeled cells and migrasomes, and allows dynamic real-time monitoring of live-cell and tissue processes. The development of HIS-SIM has further advanced the study of complex cellular organelles by providing high spatiotemporal resolution^[47,48]^. Cui *et al*. designed a small-molecule fluorescent probe, TTCPy, for migrasome imaging in HIS-SIM. Owing to its bright fluorescence and excellent photostability, TTCPy enables superresolution imaging of migrasomes with markedly improved spatial resolution^[46]^. HIS-SIM [Figure 2A], SEM [Figure 2B], and TEM [Figure 2C] were used to observe migrasome production in metastatic liver cancer cells^[49]^.

**Figure 2:**
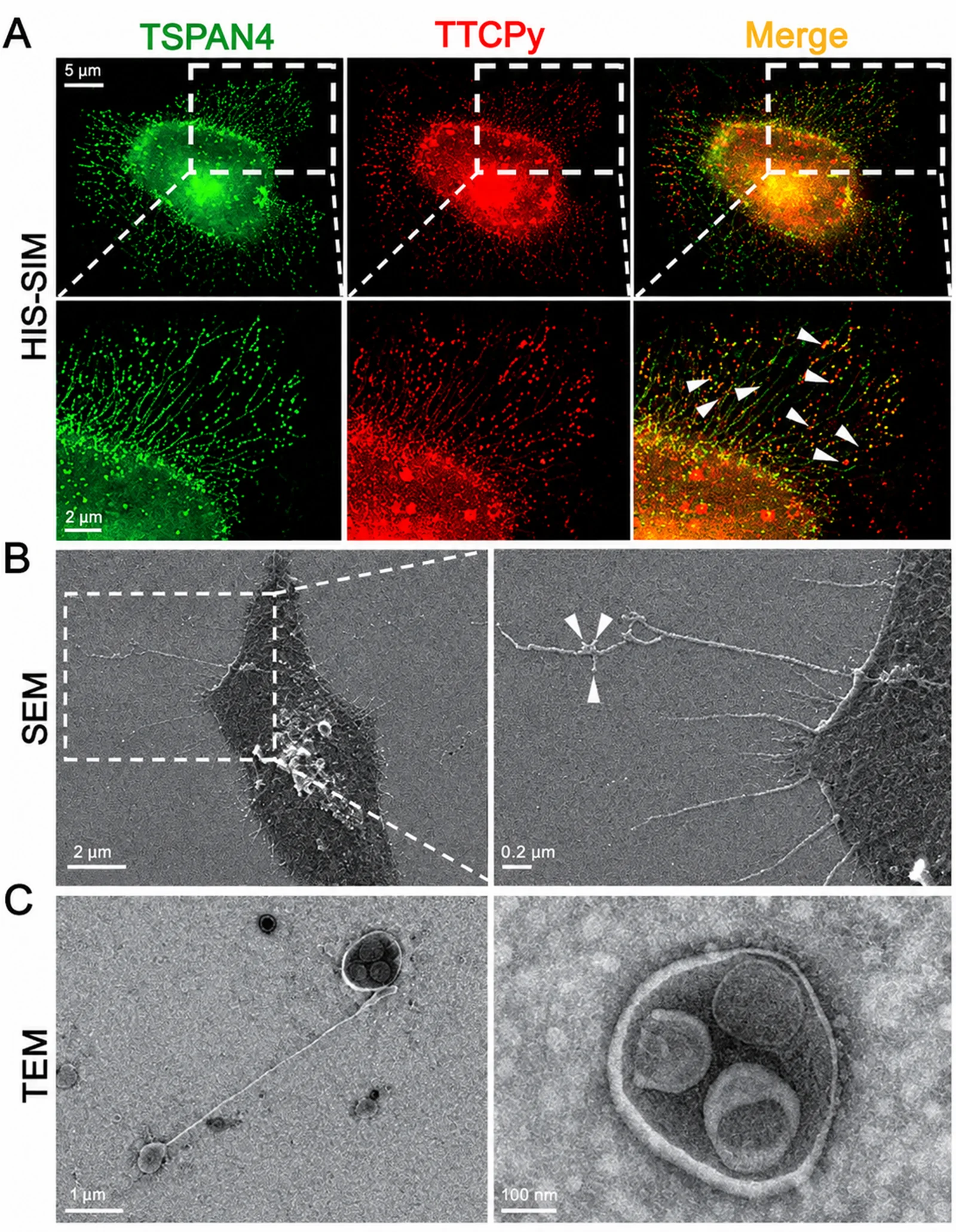
Morphological characterization of migrasomes. Representative (A) HIS-SIM (CSR Biotech, Guangzhou); (B) SEM (JSM-7900F, JEOL, Japan); and (C) TEM (JEM-1400, Japan) images showing migrasomes released from metastatic liver cancer cells (MHCC-97H). Arrows indicate the migrasomes^[49]^. HIS-SIM: High-sensitivity structured illumination microscopy; SEM: scanning electron microscopy; TEM: transmission electron microscopy; TSPAN4: tetraspanin 4; TTCPy: small-molecule fluorescent probe.

#### Migrasome-associated markers

In addition to morphological evaluation, migrasome-associated markers can be detected via Western blotting. Several proteins, including tetraspanin 4 (TSPAN4), PIGK, EOGT, and ITGA5, have been identified in migrasomes^[3]^. Professor Yu’s group demonstrated that TSPAN4 is highly enriched during migrasome formation and is commonly used as a migrasome-associated marker^[1]^. TSPAN4 preferentially accumulates on highly curved membrane structures, and regulates the membrane expansion stage during migrasome biogenesis, thereby stabilizing the expanding membrane protrusions and facilitating their transformation into migrasomes. PIGK, a component of the glycosylphosphatidylinositol (GPI) transamidase complex, is associated with migrasome enrichment and tumor progression. EOGT, an endoplasmic reticulum (ER)-resident protein, facilitates Notch receptor-ligand interactions by catalyzing the (O)-linked (N)-acetylglucosamine (O-GlcNAc) modification of EGF repeats in Notch receptors and promotes migrasome formation. ITGA5 is also significantly enriched in migrasomes and contributes to migrasome biogenesis.

#### Particle tracking analysis

Particle tracking analysis, including nanoparticle tracking analysis (NTA) and dynamic light scattering (DLS), has been employed for particle size distribution analysis of migrasomes^[50,51]^. However, discrepancies exist between the migrasome sizes measured via these two techniques and those measured via the classical methods. Lee *et al*. reported a mean migrasome diameter of 307.7 ± 6.0 nm via NTA in glioblastoma (GBM) cells^[50]^, which is substantially smaller than the 500-3,000 nm range observed via TEM and SEM^[1,8,13]^. Similarly, Gu *et al*. detected human umbilical cord mesenchymal stem cell (hUC-MSC)-derived migrasomes with a predominant peak below 500 nm by NTA, alongside a minor population exceeding 500 nm^[51]^. These discrepancies likely arise from several technical factors: (1) Detection limits: most commercial NTA instruments have optimal detection ranges of 50-1,000 nm, and may underestimate larger particles owing to Brownian motion tracking limitations; (2) Shearing forces: Sample preparation (pipetting, filtration) may fragment larger migrasomes into smaller vesicles; (3) Heterogeneity: migrasome populations are inherently heterogeneous. NTA may detect smaller MDNP-containing migrasomes or migrasome fragments while missing larger intact structures; (4) Optical properties: the refractive index assumption used by NTA software may not accurately reflect the migrasome membrane composition. Zeta potential measurements have shown that migrasomes carry a negative surface charge, which is consistent with their phospholipid bilayer composition^[49,52]^. Thus, size determination of migrasomes should not rely solely on NTA or DLS. The combined multimodal approaches include (1) electron microscopy (TEM/SEM) for direct visualization of intact migrasome morphology; (2) NTA for population-level size distribution analysis; and (3) fluorescence nanoparticle tracking analysis (fNTA) based on TSPAN4-green fluorescent protein (GFP) transfection or equivalent migrasome-exclusive labeling, which provides the most comprehensive size characterization.

#### Other characterization methods

In addition to conventional morphological and marker-based approaches [Table 2], several advanced characterization methods have been applied to migrasome analysis. Quantitative proteomics, including tandem mass tag (TMT)-labeled and label-free mass spectrometry, has been applied to identify migrasome-associated markers (NDST1, PIGK, EOGT, CPQ, *etc*.), revealing that migrasomes and exosomes share only 27% of their protein content^[13]^. MicroRNA sequencing of migrasomes and MDNPs has demonstrated distinct microRNA (miRNA) cargo profiles compared with those of exosomes, with MDNPs carrying unique microRNA species potentially involved in intercellular communication^[3]^. In addition, WGA staining has emerged as a convenient method for detecting migrasomes in fixed and living cells^[24]^.

**Table 2:** Minimal criteria for migrasome identification

**Criterion**	**Requirements**
Morphology	Pomegranate-like vesicular structures (0.5-3 µm diameter) containing smaller internal vesicles (50-100 nm); visible by TEM, SEM, or high-resolution fluorescence microscopy
Retraction fiber association	Associated with RFs at the time of formation; may be assessed by live-cell imaging or by detection of RF-associated proteins (e.g., Vimentin, F-actin)
Positive markers	TSPAN4, TSPAN7, ITGA5, ITGB1, NDST1, PIGK, EOGT, or CPQ
Negative markers	Absence or minimal presence of exosome markers (CD63, CD81, TSG101)
Functional association	Migration-dependent formation; demonstrable in wound healing, transwell migration, or equivalent assays
Biochemical validation	Confirmation by Western blotting for migrasome-associated proteins; Feasible, quantitative proteomics showing their enrichment relative to exosomes
Imaging validation	At minimum: (1) fluorescence microscopy imaging of TSPAN4-GFP or equivalent migrasome marker; and (2) TEM or SEM confirming pomegranate-like ultrastructure

CD63: Cluster of differentiation 63; CD81: cluster of differentiation 81; CPQ: carboxypeptidase Q; EOGT: EGF domain-specific O-linked N-acetylglucosamine transferase; F-actin: filamentous actin; GFP: green fluorescent protein; ITGA5: integrin alpha 5; ITGB1: integrin beta 1; NDST1: N-deacetylase-N-sulfotransferase 1; PIGK: phosphatidylinositol glycan, class K; RFs: retraction fibers; SEM: scanning electron microscopy; TEM: transmission electron microscopy; TSG101: tumor susceptibility gene 101; TSPAN4: tetraspanin 4; TSPAN7: tetraspanin 7.

## MIGRASOMES RESHAPE THE TME

The TME, which is composed of immune cells, nearby blood vessels, fibroblasts, various signaling molecules, and the ECM, plays a significant role in tumor progression. The TME is involved in many key processes, including immune regulation, ECM remodeling, and angiogenesis. Migrasomes are increasingly recognized as having important interactions within this microenvironment [Figure 3].

**Figure 3:**
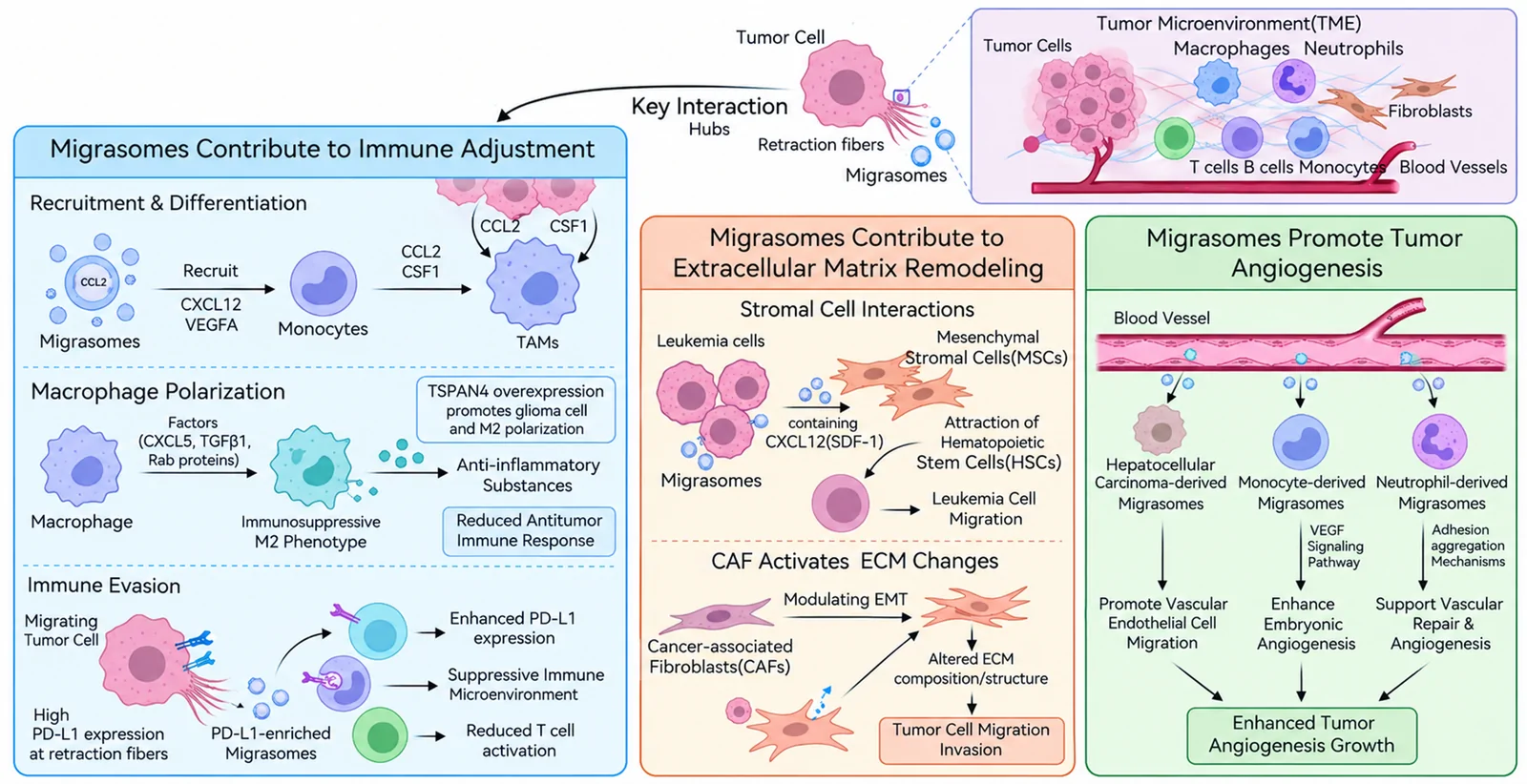
Migrasomes reshape the tumor microenvironment. Migrasomes modulate key processes within the TME, including immune regulation, ECM remodeling, and angiogenesis, by altering tumor cell behavior and reprogramming intercellular interactions. CAFs: Cancer-associated fibroblasts; CCL2: C-C motif chemokine ligand 2; CSF1: colony-stimulating factor 1; CXCL12: C-X-C motif chemokine ligand 12; CXCL5: C-X-C motif chemokine ligand 5; ECM: extracellular matrix; EMT: epithelial-mesenchymal transition; HSCs: hematopoietic stem cells; MSCs: mesenchymal stromal cells; PD-L1: programmed death-ligand 1; SDF-1: stromal cell-derived factor 1; TAMs: tumor-associated macrophages; TGFβ1: transforming growth factor beta 1; TME: tumor microenvironment; TSPAN4: tetraspanin 4; VEGF: vascular endothelial growth factor; VEGFA: vascular endothelial growth factor A.

### Migrasomes contribute to immune regulation

Migrasomes contribute to immune regulation in the TME by interacting with numerous immune cells. Various macrophage subtypes, mast cells, neutrophils, T cells, and B cells play important roles in tumor development^[53-55]^. These interactions modulate immune activation and influence antitumor immunity. Monocytes have been reported to contribute to tumor growth. Studies have shown that migrasomes recruit monocytes and promote angiogenesis through the release of C-X-C motif chemokine ligand 12 (CXCL12) and vascular endothelial growth factor A (VEGFA)^[6]^. In tumor tissues, the overexpression of angiogenic factors such as VEGFA contributes to tumor angiogenesis and promotes tumor progression^[56,57]^.

Pancancer data analysis revealed a significant positive correlation between the abundance of macrophages in the TME and the migrasome score across most tumors^[9]^. Monocytes and macrophages are among the most prevalent immune cells in the TME, accounting for more than 40% of its major cellular components. Approximately 70%-80% of myeloid cells infiltrating tumors are macrophages^[58]^. Migrasomes promote macrophage polarization toward the immunosuppressive M2 phenotype by releasing bioactive factors or inducing autophagy. M2 macrophages inhibit the antitumor immune response by releasing anti-inflammatory mediators^[29,59]^. Wang *et al*. reported that elevated expression of TSPAN4, a key regulator of migrasome formation, was associated with short overall survival across multiple cancer types. TSPAN4 expression is also correlated with genomic heterogeneity, stemness, and the TME^[60]^. TSPAN4 overexpression promoted glioma cell proliferation and induced macrophage polarization toward an M2 phenotype, suggesting its involvement in shaping an immunosuppressive TME^[60]^. In addition, tumor cells secrete C-C motif chemokine ligand 2 (CCL2) and colony-stimulating factor 1 (CSF1) which recruit monocytes from peripheral circulation into the TME, where they differentiate into tumor-associated macrophages (TAMs)^[61,62]^. One study demonstrated that pancreatic cancer cells produce migrasomes enriched with C-X-C motif chemokine ligand 5 (CXCL5), transforming growth factor beta 1 (TGFβ1), integrin-associated proteins, and Rab family proteins. These factors can induce macrophage M2 polarization, thereby suppressing inflammation and promoting tumor progression, ultimately contributing to the formation of an immunosuppressive TME^[29,63]^.

Emerging evidence indicates that migrasomes serve as important mediators of immune evasion in the TME. Migrasomes can package and deliver immune checkpoint ligands, such as programmed death-ligand 1 (PD-L1), to neighboring immune cells, including T cells, natural killer (NK) cells, and macrophages. Following internalization, these ligands suppress antitumor immunity by inducing apoptosis, inhibiting immune cell proliferation, and promoting macrophage polarization toward protumorigenic phenotypes, thereby helping tumor cells evade immune surveillance. Consistent with these findings, one study reported that PD-L1 facilitates directionally persistent cancer cell migration by regulating the localization, cytoskeletal association, and signaling properties of the β4 integrin. PD-L1 concentrates at the rear of migrating cancer cells where it results in the formation of PD-L1-containing RFs and migrasomes. During migration, terminal retraction leads to the release of PD-L1-containing migrasomes^[64]^. After uptake by surrounding cells, PD-L1-enriched migrasomes further increase PD-L1 expression, contributing to an immunosuppressive microenvironment and weakening antitumor immune responses. In addition, these migrasomes release chemokines that facilitate breast cancer cell migration within the TME, thereby increasing metastatic potential^[65]^.

### Migrasomes foster extracellular matrix remodeling

Migrasomes significantly influence stromal cell behavior and interactions between tumor cells and the ECM. The ECM plays a crucial role in tumor progression by providing a molecular framework that supports biomechanical activities and cellular functions^[66]^. Mesenchymal stromal cells (MSCs), which are precursors to multiple cell types, can generate migrasomes and thereby participate in intercellular communication within the TME.

In hematopoietic niches and leukemias, the CXCL12-CXCR4 (C-X-C motif chemokine receptor 4) axis is a key regulator of hematopoietic stem and progenitor cell trafficking and leukemia cell homing, and MSCs are a major source of CXCL12 in the bone marrow. Recent work has shown that MSC-derived migrasomes are enriched with CXCL12 and promote the migration of hematopoietic stem and progenitor cells via CXCR4 signaling, suggesting that migrasomes may facilitate interactions between leukemia cells and bone marrow stromal cells, thereby promoting cancer cell migration^[67]^. Migrasomes also promote the osteogenic differentiation of MSCs and enhance macrophage phagocytosis and M2 polarization in tissue repair and infectious or inflammatory contexts, indicating broader roles in tissue homeostasis and immune regulation^[68]^. These findings underscore the contributions of MSCs and migrasomes to the TME^[69]^. In addition, migrasomes have been implicated in the activation of cancer-associated fibroblasts (CAFs) and in the modulation of epithelial-mesenchymal transition (EMT) programs in cancer cells within the TME. By altering the ECM composition, organization, and biochemical signaling, migrasome-mediated stromal reprogramming can subsequently influence tumor cell migration, invasion, and metastatic dissemination^[30,70]^.

### Migrasomes promote tumor angiogenesis

Migrasomes contribute to tumor angiogenesis and influence tumor vascularization in the TME. In hepatocellular carcinoma (HCC) models, vascular endothelial growth factor (VEGF)-enriched migrasomes facilitate the migration of vascular endothelial cells and promote angiogenesis and HCC progression^[71]^. Another study identified a subset of POSTN^+^ CAFs, which are predominantly located at the tumor-stroma interface, as the main source of migrasomes in HCC. POSTN^+^ CAF-derived migrasomes enhance endothelial angiogenic activity by delivering VEGFA and activating the vascular endothelial growth factor receptor 2 (VEGFR2)-phosphatidylinositol 3-kinase (PI3K)-protein kinase B (AKT)-endothelial nitric oxide synthase (eNOS) pathway, promoting vascular remodeling within HCC^[72]^. These findings revealed that migrasomes derived from different origins act as key drivers of HCC progression by promoting angiogenesis. In addition, previous studies have indicated that migrasomes released by monocytes also increase embryonic angiogenesis, at least in part through activation of the vascular endothelial growth factor (VEGF) signaling pathway^[6,73]^. Furthermore, migrasomes derived from neutrophils have been shown to support vascular repair and angiogenesis following tissue injury via adhesion-dependent aggregation mechanisms^[43]^. Collectively, these findings support the concept that migrasomes play an important role in regulating angiogenesis, including within the TME, potentially through classical VEGF signaling.

## MIGRASOMES EXPEDITE CANCER METASTASIS AND PROGRESSION

Cell migration, a key feature of cancer malignancy, is crucial for cancer progression and metastasis. The production of migrasomes is tightly linked to the movement of migrating cells, as they form on RFs during cell migration. Migrasomes are therefore likely to participate in the biological activities of migrating cells, including tumor invasion and metastatic spread. Thus, it is reasonable to speculate that migration-dependent migrasomes may represent novel players in mediating cancer development and dissemination [Figure 4].

**Figure 4:**
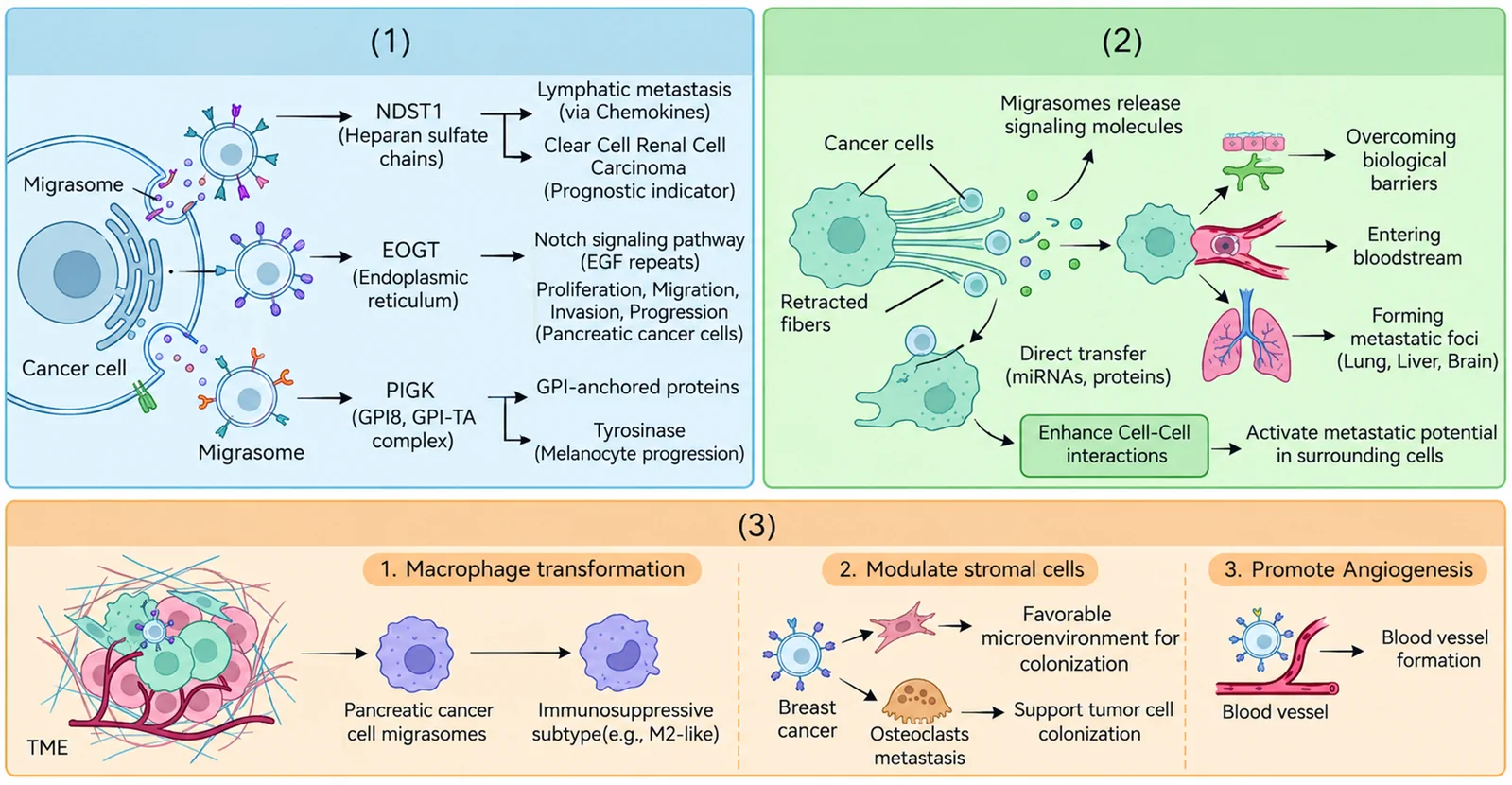
Migrasomes expedite cancer metastasis and progression. (1) Migrasome-associated markers modulate cancer cell invasion, migration, and metastatic spread; (2) Migrasomes release signaling molecules to neighboring cells, increasing their motility and pro-metastatic behavior; (3) Migrasomes help establish an immunosuppressive TME that supports tumor progression and colonization. EGF: Epidermal growth factor; EOGT: EGF domain-specific O-linked N-acetylglucosamine transferase; GPI: glycosylphosphatidylinositol; GPI-TA: glycosylphosphatidylinositol transamidase; miRNAs: microRNAs; NDST1: N-deacetylase-N-sulfotransferase 1; PIGK: phosphatidylinositol glycan, class K; TME: tumor microenvironment.

### Migrasome-associated markers in cancer metastasis and progression

To date, functional studies involving migrasome-associated marker genes within the context of migrasome biology are limited [Table 3]^[29,50,60,64,65,71,72,74-77]^. Most studies have explored the role of migrasomes in multiple cancer processes by analyzing the associations between ‘migrasome genes’ and malignancies^[9]^. Mutations or altered expression of migrasome-related genes (MRGs), such as *NDST1*, *EOGT*, *PIGK, ITGB1*, *ITGA5*, *CPQ* and *TSPAN4,* have been correlated with tumor mutational burden and microsatellite instability. These migrasome-associated markers also influence cancer progression through their broader roles in cell signaling, adhesion, and matrix interactions. However, notably, these genes are also present in other EV populations and are broadly implicated in cancer biology; therefore, these correlations do not establish migrasome-specific functions in cancer progression.

**Table 3:** Summary of migrasome-associated markers in cancers

**Cancer type**	**Migrasome source**	**Markers used**	**Key findings**	**Evidence type**	**Ref.**
Hepatocellular carcinoma	Cancer cells; POSTN^+^ CAFs	CD151, TSPAN4	Promote angiogenesis, invasion; induce immunotherapy resistance	Direct migrasome evidences; Gene-association evidences	[71,72]
Pancreatic cancer	Cancer cells	TSPAN4, CXCL5, TGFβ1	M2 macrophage polarization; immunosuppressive TME; gemcitabine resistance	Direct migrasome evidences	[29]
Glioblastoma	Cancer cells	TSPAN4	Promote cell proliferation; M2 polarization; EGFR stability regulation	Direct migrasome evidences	[50,60,76]
Esophageal cancer	Cancer cells	TSPAN4	Paclitaxel resistance via apoptosis inhibition	Direct migrasome evidences	[77]
Breast cancer	Cancer cells	TSPAN4, PD-L1	PD-L1-enriched migrasomes promote immune evasion	Direct migrasome evidence	[64,65]
Gastric cancer	-	Tetraspanin family members	Central hub genes identified via bioinformatics	Bioinformatic and experimental evidences	[75]
Renal cell carcinoma	-	NDST1	Prognostic signature; associated with tumor angiogenesis	Bioinformatic evidences	[74]
Multiple cancer types	-	TSPAN4, NDST1, EOGT, PIGK, CPQ, ITGA5, ITGB1	Migrasome score correlates with immune score, stroma score, prognosis	Bioinformatic and experimental evidences	[60]

CAFs: Cancer-associated fibroblasts; CD151: cluster of differentiation 151; CPQ: carboxypeptidase Q; CXCL5: C-X-C motif chemokine ligand 5; EGFR: epidermal growth factor receptor; EOGT: EGF domain-specific O-linked N-acetylglucosamine transferase; ITGA5: integrin alpha 5; ITGB1: integrin beta 1; M2: M2-polarized macrophage phenotype; NDST1: N-deacetylase-N-sulfotransferase 1; PD-L1: programmed death-ligand 1; PIGK: phosphatidylinositol glycan, class K; POSTN: periostin; TGFβ1: transforming growth factor beta 1; TME: tumor microenvironment; TSPAN4: tetraspanin 4.

NDST1 participates in the biosynthesis of heparan sulfate (HS) chains, which can modulate chemokine gradients and thereby contribute to lymphatic metastasis as mediators of chemokine action. Zeng *et al*. identified NDST1 as a potential prognostic marker in clear cell renal cell carcinoma, and associated its expression with patient outcomes^[74]^. Recent NDST1-focused works have examined its function in diverse aspects of human cancer, including T-cell-mediated antitumor immunity, tumor growth, and tumor angiogenesis^[78-80]^. Fuster *et al*. reported that deletion of *NDST1* in lymphatic endothelial cells impaired tumor cell migration, and that *NDST1* mutation selectively inhibited tumor angiogenesis without substantially affecting physiological angiogenesis, suggesting a degree of tumor specificity^[80]^. In breast cancer, Okolicsanyi *et al*. reported that the HS agonist heparin increased *NDST1* expression, activated WNT signaling, and promoted tumor cell proliferation and migration^[81]^, whereas NDST1 knockdown suppressed HS production and reduced renal carcinoma cell invasion, metastasis, and migration^[82]^. EOGT, an ER-localized protein, modifies EGF-like repeats on Notch receptors and thereby regulates Notch signaling. Through this mechanism, EOGT can promote the proliferation, migration, and invasion of pancreatic cancer cells, thereby driving cancer progression^[83]^. Barua *et al*. combined bioinformatics and functional assays and reported that EOGT affects the growth and motility of pancreatic cancer cells and influences overall survival in patients^[84]^. In HCC, EOGT expression is significantly higher than that in normal liver tissue and is associated with immune infiltration, advanced tumor stage, and reduced overall survival, highlighting its prognostic relevance^[85]^. PIGK is an essential component of the glycosylphosphatidylinositol transamidase (GPI-TA) complex, which anchors proteins to GPI and thereby regulates their cell surface expression^[86]^. Dysregulated PIGK expression has been linked to malignancies, and its inhibition can alter tyrosinase expression or function, potentially impacting melanocyte biology and melanoma progression^[87]^. TSPAN4, a key migrasome structural protein, is aberrantly expressed in multiple cancers, and is closely linked to its methylation status, tumor heterogeneity, stemness, and suppressive immune microenvironment^[14,61,88,89]^. Kim *et al*. analyzed bioinformatics datasets and reported that TSPAN4 is significantly upregulated in gastric cancer tissues compared with adjacent normal tissues and promotes tumor formation in xenografts^[78]^. El Ghazal *et al*. demonstrated that high TSPAN4 expression is correlated with poor prognosis in GBM patients. TSPAN4 knockdown dramatically inhibits proliferation, invasion, and *in vivo* tumorigenicity, whereas TSPAN4 overexpression accelerates GBM progression by activating the EGFR signaling pathway, suggesting that therapeutic targeting of TSPAN4 may confer substantial clinical benefits for GBM patients^[79]^. In pancreatic cancer, TSPAN4^+^ fibroblasts are located predominantly at the tumor periphery, and display elevated migrasome-related gene expression. TSPAN4^+^ fibroblasts engage in extensive crosstalk with immune and endothelial cells to facilitate metastasis and immune evasion, which is associated with enhanced ECM remodeling and immune suppression^[88]^.

Together, these studies suggest that genes associated with migrasome biogenesis or function can influence cancer metastasis and progression, although many of these effects are mediated through broader signaling and adhesion pathways rather than migrasomes alone. Future direct experimental evidence based on migrasome-specific isolation and functional assays is needed to establish the causal relationships of migrasome-associated markers with cancer metastasis and progression.

### Migrasomes release signaling molecules to surrounding cells

During metastasis, migrasome-mediated intercellular communication helps cancer cells overcome biological barriers, intravasate, survive in the circulation, and ultimately form metastatic foci in distant organs such as the lungs, liver, and brain. By releasing chemokines, growth factors, and other signaling molecules, migrasomes can modify the behavior of neighboring cancer and stromal cells, thereby enhancing cell-cell interactions and increasing their metastatic potential^[90]^. Additionally, migrasomes may directly transfer microRNAs and proteins to recipient cancer cells, thereby increasing their invasive and migratory capabilities^[91]^. In GBM cells, migrasomes reportedly contribute to tumor spread by transporting migration-associated signaling molecules and reorganizing communication networks that support local invasion and distant dissemination^[60,92]^.

### Migrasomes facilitate the immunosuppressive microenvironment

Interactions between tumor cells and the TME are essential for metastasis. The TME comprises fibroblasts, immune and inflammatory cells, blood and lymphatic vessels, and ECM components^[93]^. Zhang *et al*. demonstrated that pancreatic cancer cells induce macrophages to adopt immunosuppressive phenotypes via migrasomes. These transformed macrophages overexpress immunosuppressive factors, such as arginase 1 (ARG-1), which inhibits CD4^+^ and CD8^+^ T-cell proliferation, thereby weakening antitumor immunity and facilitating cancer cell migration and invasion^[29]^. Thus, migrasomes contribute to the establishment of an immunosuppressive TME by modulating myeloid cell function and T-cell responses. Migrasomes also regulate stromal cells, thereby shaping premetastatic niches. In breast cancer-associated bone metastasis, osteoclasts remodel the bone microenvironment to support tumor cell colonization. Migrasomes have been implicated in this process by mediating communication between tumor cells and osteoclasts, thereby promoting a bone milieu that favors metastatic seeding and growth^[10,94]^. Furthermore, migrasomes can promote tumor metastasis by supporting angiogenesis within the TME, for example, through proangiogenic cargos and interactions with endothelial and myeloid cells^[6,70]^. Collectively, these observations indicate that migrasomes play important roles in cancer progression and metastasis by integrating migratory signaling, stromal reprogramming, immune suppression, and neovascularization.

## MIGRASOMES CONFER DRUG RESISTANCE IN CANCER

Cancer drug resistance remains a significant challenge in oncology, particularly in the context of chemotherapy and targeted therapies, and is a primary cause of therapeutic failure. The interaction between migrasomes and drug resistance pathways in cancers is an emerging research focus [Figure 5]. It is increasingly recognized that migrasomes may modulate both intrinsic and microenvironmental resistance mechanisms in tumors.

**Figure 5:**
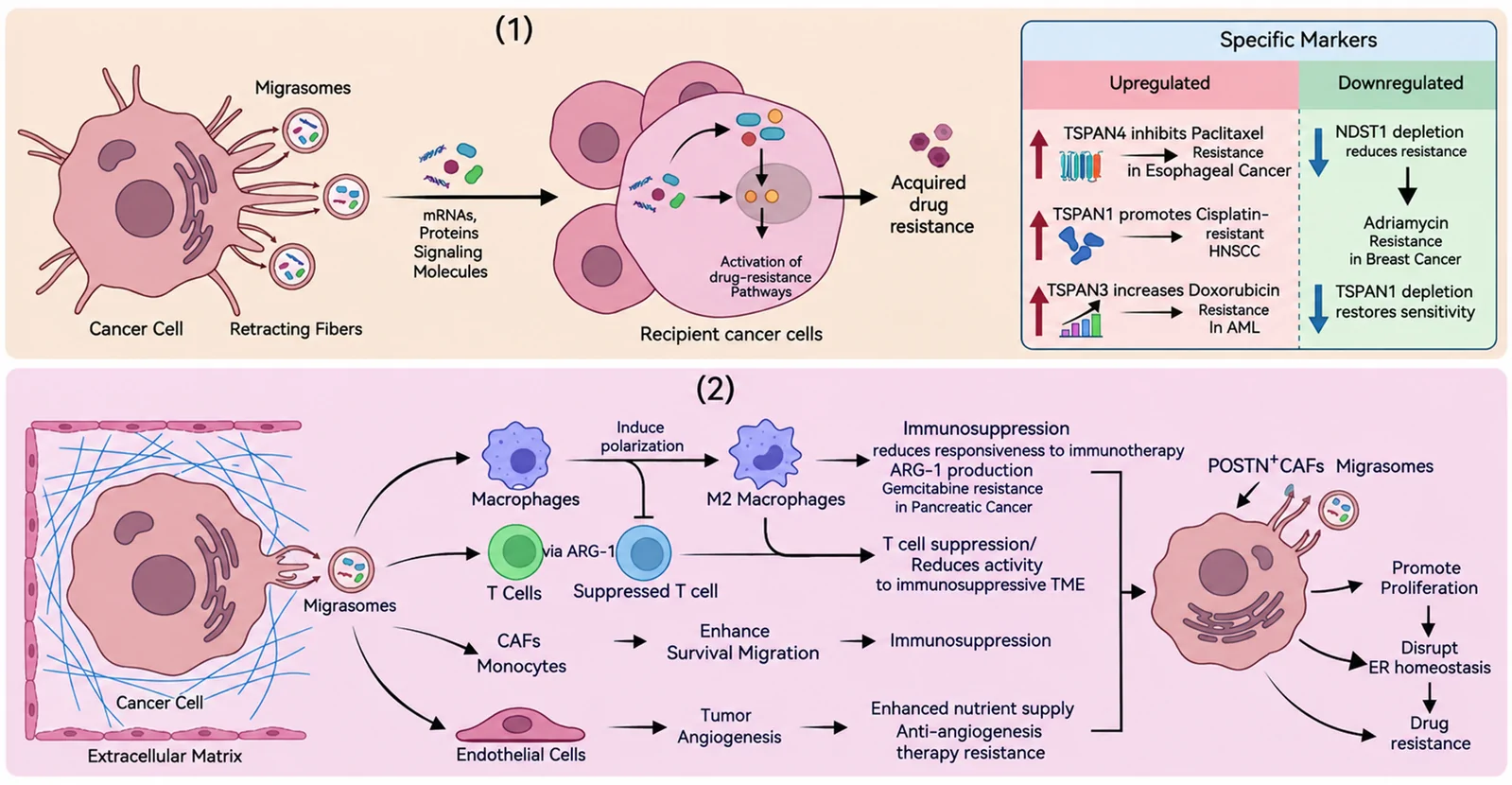
Migrasomes confer drug resistance in cancer. (1) Migrasomes transfer resistance-associated signaling molecules (such as RNAs and proteins) to neighboring cancer cells, increasing their survival and drug resistance phenotypes; (2) Migrasomes remodel the TME by promoting immunosuppressive cell states and angiogenesis, thereby creating a niche that supports cancer drug resistance. AML: Acute myeloid leukemia; ARG-1: arginase 1; CAFs: cancer-associated fibroblasts; ER: endoplasmic reticulum; HNSCC: head and neck squamous cell carcinoma; mRNAs: messenger RNAs; NDST1: N-deacetylase-N-sulfotransferase 1; POSTN: periostin; TME: tumor microenvironment; TSPAN1: tetraspanin 1; TSPAN3: tetraspanin 3; TSPAN4: tetraspanin 4.

### Migrasomes impart drug resistance via the transfer of signaling molecules

Direct evidence specifically linking migrasome-carried signaling molecules to drug resistance in cancers remains limited. Migrasomes carry diverse signaling molecules that influence drug resistance pathways. By delivering mRNAs, proteins, and other bioactive cargos, migrasomes stimulate surrounding cancer cells to acquire or enhance resistance phenotypes^[90,91]^. In addition, several migrasome-associated markers have been implicated in drug resistance. For example, TSPAN4 has been reported to facilitate paclitaxel resistance by inhibiting apoptosis in esophageal cancer, suggesting that TSPAN-enriched membrane microdomains may contribute to chemoresistance^[77]^. Notably, in this study, TSPAN4 overexpression in cancer cells was examined in general, and was not specific to migrasomes. The migrasome-associated enzyme NDST1 has also been linked to anthracycline (e.g., adriamycin) resistance in breast cancer, likely through its role in HS biosynthesis and the modulation of growth factor and chemokine signaling^[95]^. Other TSPAN family members enriched in migrasomes, such as tetraspanin 1 (TSPAN1) and tetraspanin 3 (TSPAN3), may similarly modulate drug resistance. In cisplatin-resistant head and neck squamous cell carcinoma, the depletion of TSPAN1 reduces proliferation and restores drug sensitivity, whereas the upregulation of TSPAN3 in doxorubicin-resistant acute myeloid leukemia enhances drug resistance^[96]^. Our recent study demonstrated that macropinocytosis of CD147-positive hypoxia-induced migrasomes (hypo-Migs) activates the PI3K/AKT/TWIST1 signaling pathway, subsequently inducing vasculogenic mimicry (VM) formation and promoting sorafenib resistance in HCC^[49]^. These findings suggest that migrasomes and their associated markers influence cancer drug resistance through both cargo transfer and signaling regulation. However, more research is needed to determine how frequently migrasome-related mechanisms operate across different cancer types and treatment regimens. Clarifying these context-dependent differences will be essential to the rational design of targeted therapies against migrasome-mediated drug resistance.

### Migrasomes confer drug resistance by regulating the TME

The TME plays a pivotal role in conferring cancer drug resistance by shaping immune evasion, angiogenesis and ECM remodeling^[97]^. Migrasomes substantially influence the TME, and thereby affect treatment responses. For example, migrasomes facilitate macrophage M2-polarization in the TME, which in turn promotes the survival, migration, and immunosuppressive functions of myeloid and stromal cells, including CAFs and monocytes^[29,98]^. These cells contribute to an immunosuppressive TME that diminishes tumor responsiveness to immunotherapy and other systemic treatments^[59,99]^. In pancreatic cancer, tumor-derived migrasomes have been shown to induce immunosuppression by promoting macrophage M2-polarization and suppressing T-cell activity via ARG-1, thereby supporting tumor growth and resistance to gemcitabine^[29]^. Migrasomes also promote tumor angiogenesis, increasing the nutrient and oxygen supply to tumor cells and potentially increasing resistance to antiangiogenic therapies^[71]^. In HCC, migrasomes derived from POSTN-positive CAFs drive tumor progression and drug resistance by promoting cancer cell proliferation and disrupting ER homeostasis^[72]^. Collectively, these data indicate that migrasomes contribute to cancer drug resistance through both direct effects on tumor cells and indirect modulation of the TME. Targeting migrasomes themselves, or the pathways they regulate in the TME, may offer promising strategies to overcome therapeutic resistance in specific cancer contexts.

## CLINICAL APPLICATION OF MIGRASOMES IN CANCERS

### Diagnosis

To date, no migrasome-based diagnostic tools have been clinically validated; however, there have been significant advances in imaging and molecular techniques that could be adapted to detect migrasomes in cancers. Although migrasomes are relatively newly recognized structures, several existing modalities have potential for visualization and molecular characterization.

Mass spectrometry imaging (MSI) has emerged as a powerful tool for spatially resolved multiomics analysis in neurology and other fields^[100]^. MSI combines the multichannel detection capability of a mass spectrometer with surface sampling to map the distribution of metabolites, lipids, and proteins in tissues. Because migrasomes contain specific proteins, lipids, and nucleic acids, MSI could be used to detect characteristic molecular components of migrasomes in tumor tissues, providing information about their location and abundance. This application remains largely conceptual at present and would require rigorous validation.

Fluorescence microscopy is another relevant imaging technique. By using specific fluorescent probes or antibodies targeting migrasome-associated proteins, such as TSPAN4, which is enriched in migrasome-like vesicles^[101]^, fluorescence microscopy can visualize migrasome formation and dynamics in real time. These findings can help researchers study the processes of migrasome biogenesis, release, and interaction with surrounding cells in tumors. However, the development of highly specific and sensitive fluorescent probes or antibodies that unambiguously distinguish migrasomes from other EVs remains a major challenge [Figure 6A].

**Figure 6:**
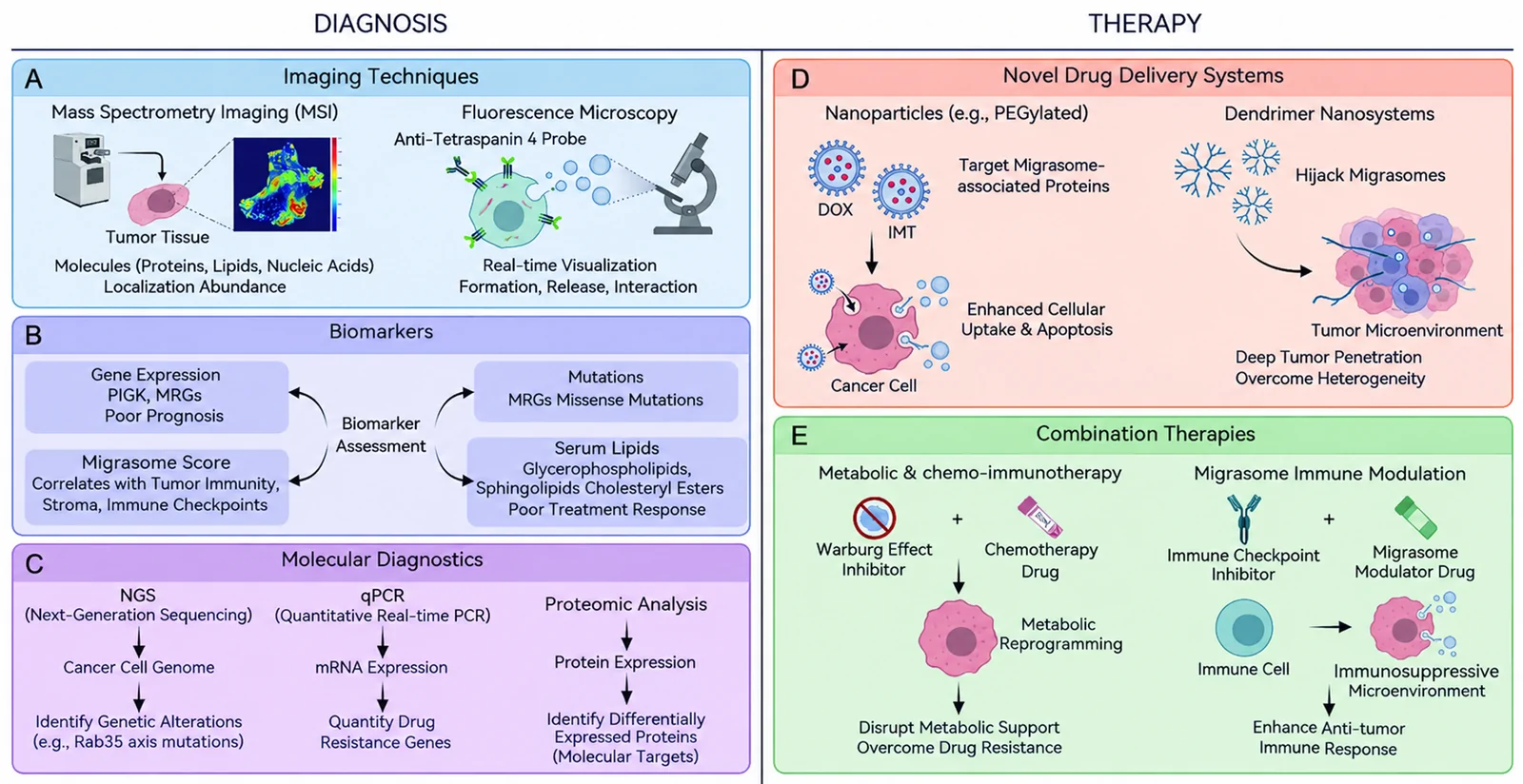
Clinical application of migrasomes in cancers (A-E). By integrating advanced imaging, molecular profiling, and targeted treatment strategies, migrasomes show substantial potential in cancer diagnosis and therapy. (A) Mass spectrometry imaging and fluorescence microscopy can be used to visualize migrasomes and assess their spatial distribution in tumors; (B) Migrasome-associated biomarkers, including MRG expression levels, missense mutations in MRGs, composite migrasome scores, and serum lipid signatures, may provide prognostic and predictive information; (C) Molecular diagnostic approaches such as next-generation sequencing, quantitative real-time PCR, and proteomic profiling enable the characterization of migrasome-associated drug resistance mechanisms; (D and E) Therapeutically, migrasome-targeted or migrasome-informed nanoparticle drug-delivery systems offer promising options for overcoming cancer drug resistance and improving treatment efficacy. DOX: Doxorubicin; IMT: imatinib; MRGs: migrasome-related genes; mRNA: messenger RNA; MSI: mass spectrometry imaging; NGS: next-generation sequencing; PIGK: phosphatidylinositol glycan, class K; qPCR: quantitative real-time PCR; Rab35: Ras-related GTP-binding protein 35.

Biomarkers are crucial for assessing migrasome-related activity and potential contributions to drug resistance in cancer. Pancancer analyses have identified candidate migrasome-associated biomarkers at the transcriptomic and genomic levels. For example, *PIGK* expression is significantly upregulated in 22 of 33 tumors, and high expression of gene sets associated with migrasome biogenesis and function has been linked to poor prognosis in several cancers. Missense mutations and copy number alterations in MRGs have also been described, suggesting that these genetic changes could serve as biomarkers of altered migrasome pathways. A composite ‘migrasome score’, derived via gene set enrichment analysis of MRGs, has been reported to correlate with tumor immune scores, stromal scores, and immune checkpoint gene expression, indicating potential utility in understanding the role of migrasomes in tumor immunity and drug resistance^[9]^. In multiple myeloma, serum lipidomics has been used to explore biomarkers predictive of the response to bortezomib therapy, with lower levels of specific glycerophospholipids, sphingolipids, and cholesteryl esters associated with poor treatment response^[102]^. Although these lipid changes are not specific to migrasomes, they intersect with migrasome-associated lipid metabolism and vesicle biology, and thus could indirectly reflect migrasome-related functions in drug resistance. This link remains hypothesis-generating and requires direct experimental confirmation [Figure 6B].

Molecular diagnostic tools also provide important information on migrasome-related drug resistance. Next-generation sequencing (NGS) can identify genetic alterations in genes involved in migrasome formation and trafficking, such as those involved in the PI(4,5)P2-Rab35 axis^[103]^. Mutations in these pathways are associated with altered migrasome function and therapy resistance^[4]^. Quantitative real-time PCR can be used to measure the expression levels of MRGs and downstream effector genes implicated in migrasome-mediated drug resistance. Similarly, studies of Leishmania tropica antimony resistance have examined genes involved in trypanothione metabolism and drug transport via quantitative real-time PCR (qPCR); similar approaches can be applied to cancer to explore migrasome-associated resistance mechanisms^[104]^. Additionally, proteomic analysis can identify proteins that are differentially expressed in migrasome-rich *vs*. migrasome-poor cancer cells or in drug-resistant *vs*. drug-sensitive cancers, providing candidate diagnostic and prognostic markers [Figure 6C].

### Therapy

Novel therapeutic strategies targeting migrasomes or exploiting migrasome biology are at an early conceptual stage but may offer opportunities to improve the efficacy of cancer treatments, particularly in resistant tumors. One area of interest is the development of drug delivery systems that can target migrasome-associated pathways. For example, PEGylated nanostructured platforms have been designed for the coadministration of doxorubicin and imatinib to inhibit metastatic tumors^[105]^, achieving sequential and sustained drug release, enhanced cellular drug uptake, and increased apoptosis. Although these systems are not specifically designed for migrasomes, similar nanocarriers could, in theory, be engineered to recognize migrasome-associated proteins or receptors on tumor cells, thereby delivering drugs to migrasome formation or action sites and disrupting migrasome-mediated resistance more effectively [Figure 6D].

Dendrimer-based nanosystems represent another promising delivery system. These structures can self-assemble into nanomicelles and interact with EVs in the TME, effectively ‘hijacking’ their trafficking pathways^[106]^. Given that migrasomes share some structural and functional features with other EVs, dendrimer nanosystems might be adapted to target migrasomes and deliver therapeutic agents to tumor and stromal cells. Such approaches could help overcome tumor heterogeneity and enhance deep tumor penetration, potentially enhancing the treatment efficacy in drug-resistant cancers [Figure 6D].

Combination therapies are also promising strategies for counteracting migrasome-associated drug resistance. One approach is to combine agents that target different aspects of migrasome biology and resistance pathways. For example, in prostate cancer, combining an inhibitor of the Warburg effect (e.g., glucose-conjugated 1,4-naphthoquinones) with standard chemotherapy could disrupt the metabolic support for migrasome-driven resistance, although direct evidence for a causal link between migrasomes and metabolic reprogramming remains limited^[107]^. Another strategy involves combining immunotherapies with drugs that modulate migrasome activity. In some cancers, migrasomes in the TME can suppress immune responses by reprogramming myeloid cells and dampening T-cell function^[11]^. In principle, agents that inhibit migrasome formation, cargo loading, or signaling could be combined with immune checkpoint inhibitors to enhance antitumor immunity, for example, by blocking the release or action of immunosuppressive molecules carried by migrasomes^[108,109]^ [Figure 6E].

### Challenges in clinical translation

Translating migrasome research into clinical practice faces several major challenges. First, our understanding of migrasome biology is still incomplete. Although substantial progress has been made in defining their structure, RF formation, and roles in intercellular communication, many aspects of migrasome biogenesis, cargo selection, turnover, and context-specific functions in cancers remain poorly understood. The precise molecular mechanisms governing migrasome formation and the rules by which they selectively package and transfer specific molecules have not yet been fully elucidated. Without a comprehensive mechanistic framework, rationally designing migrasome-targeted diagnostics or therapies is difficult^[11]^. Second, the development of reliable, specific, and standardized diagnostic tools for detecting migrasomes in clinical samples is still in its infancy. There are currently no widely accepted consensus markers or assays for the routine detection of migrasomes in patient tissues or body fluids. Imaging methods and candidate biomarkers are mostly at the experimental stage and require further optimization, interlaboratory validation, and clinical correlation. Third, well-designed, large-scale clinical studies are needed to evaluate the safety, feasibility, and efficacy of migrasome-targeted approaches. Such trials are complex, time-consuming, and expensive, particularly when conducted in parallel with ongoing efforts to standardize migrasome detection and quantification [Figure 6]. Moreover, disentangling the specific contributions of migrasomes from other EVs and overlapping signaling pathways will be key conceptual and practical hurdles.

Together, these challenges underscore that, while migrasomes hold considerable promise as diagnostic markers and therapeutic targets, substantial basic and translational research is still needed before migrasome-based strategies can be robustly integrated into clinical oncology.

## CONTROVERSIES AND CHALLENGES

Despite significant advances, several controversies and unresolved questions remain in migrasome research: (1) Are migrasomes distinct from other large EVs in cancer samples? A major challenge is definitively distinguishing migrasomes from large oncosomes (0.5-5 µm), apoptotic bodies (1-5 µm), and microvesicles (100-1,000 nm) in cancer samples. All these populations overlap with migrasomes in size and share surface markers^[2]^. Current evidence supporting migrasomes as a distinct entity relies primarily on their unique pomegranate-like morphology, retraction of fiber associations during formation, and the presence of certain markers (NDST1, PIGK, EOGT, CPQ) that are absent from exosomes^[13]^. However, in complex biological fluids or tumor tissues where RFs are not observable, confident identification remains difficult. Whether migrasomes represent a genuinely distinct biogenesis pathway or a specialized subtype of large EVs remains an open question requiring further investigation; (2) Are migrasome-associated markers specific? While TSPAN4, integrins, and four specific markers (NDST1, PIGK, EOGT, CPQ) are enriched in migrasomes, they are not entirely exclusive. TSPAN4 and integrins are also detected in exosomes and other EV subtypes, albeit at lower levels^[2,13,14]^. Conversely, “migrasome-specific” markers may be present in nonvesicular protein complexes or membrane fragments. The lack of a single, unambiguous migrasome marker complicates both research and clinical applications; (3) Are the observed phenotypes migrasome-dependent? Many studies linking migrasomes to cancer phenotypes rely on correlational analyses of migrasome-marker gene expression or manipulations (e.g., TSPAN4 overexpression/knockdown) that may affect multiple cellular processes beyond migrasome formation^[9,60,110]^. Direct evidence demonstrating that the observed phenotypes are specifically mediated by migrasomes remains limited. Rescue experiments combining migrasome inhibition with exogenous migrasome supplementation are needed to establish causality; (4) How does migrasome biology differ from EV-mediated communication? Migrasomes and exosomes appear to have partially overlapped but also distinct functions in intercellular communication^[2]^. Both can transfer proteins, RNAs, and lipids to recipient cells; however, migrasomes uniquely contain larger cargo (including damaged mitochondria and organelles) and form through migration-dependent mechanisms^[3,5,42]^. Whether migrasomes and exosomes operate through common downstream signaling pathways or have distinct receptor-mediated uptake mechanisms is largely unknown. Furthermore, the recent discovery of MDNPs highlights the distinction between migrasomes and exosomes^[3]^, raising questions about whether MDNPs represent a migrasome-specific EV subtype or an isolation artifact.

## CONCLUSION AND PERSPECTIVE

Migrasome research provides novel insights into the mechanisms of cell-cell communication, which generally occur through direct contact, ligand-receptor interactions, and the exchange of EVs^[91]^. Like EVs, migrasomes are enriched in nucleic acids, proteins, and lipids, which play crucial roles in intercellular signaling. Migrasomes can directly interact with neighboring cells and deliver bioactive molecules or organelles that recipient cells can internalize, therefore mediating the exchange of materials and information. Furthermore, migrasomes are enriched in signaling molecules and can enhance communication via ligand-receptor interactions^[111]^. Accumulating evidence links migrasomes to the pathology of several cancers, including HCC, GBM, gastric cancer, renal carcinoma, oral leukoplakia, and esophageal cancer^[11,17,71,75,76,112-115]^.

Although direct evidence remains limited, a growing body of indirect evidence suggests that migrasomes may play crucial roles in cancer metastasis and drug resistance, and may offer avenues for clinical application. In the context of metastasis and progression, migrasomes contribute to immune evasion by shaping the immunosuppressive TME and influencing ECM remodeling. They also promote tumor angiogenesis, at least in part via VEGF-related signaling, thereby facilitating tumor growth and dissemination. The molecular mechanisms underlying migrasome-associated drug resistance involve both tumor-intrinsic and microenvironmental pathways: first, the transfer of resistance-associated RNAs and proteins confers cell-autonomous resistance; second, migrasomes remodel the TME through M2-polarization of macrophages, immune suppression, and enhanced angiogenesis, which can diminish the efficacy of immunotherapies and antiangiogenic agents. Clinically, migrasomes show promise as diagnostic or prognostic biomarkers, and potential therapeutic targets, although this remains largely at a conceptual and preclinical stage. Despite current challenges in standardizing detection methods and validating their clinical utility, a deeper understanding of migrasome biology will be essential for translating these insights into targeted interventions.

In the future, migrasomes represent a promising and innovative frontier in cancer research. Their distinctive biological characteristics may enable advances in cancer diagnostics, particularly through their potential use as liquid biopsy biomarkers that reflect dynamic cellular states. Therapeutically, engineered migrasomes or migrasome-inspired vesicles could be developed as targeted delivery systems for drugs or genetic material, complementing existing EV-based strategies. As research progresses, migrasomes have the potential to provide novel therapeutic approaches not only in cancer but also in other diseases characterized by dysregulated intercellular communication, including developmental disorders, immune dysregulation, and infectious diseases.

## DECLARATIONS

### AI and AI-assisted tools statement

During the preparation of this manuscript, the AI tool PicDoc (version 1.17, released 2025-09-26; https://edit.picdoc.cn/dashboard) was used solely for the creation of the Graphical Abstract and Figures 1, 3, 4, 5, and 6. The tool did not influence the study design, data collection, analysis, interpretation, or the scientific content of the work. All authors take full responsibility for the accuracy, integrity, and final content of the manuscript.

### Copyright

© The Author(s) 2026.
